# Atomic Structures of Coxsackievirus B5 Provide Key Information on Viral Evolution and Survival

**DOI:** 10.1128/jvi.00105-22

**Published:** 2022-04-20

**Authors:** Peng Yang, Dawei Shi, Jianmeng Fu, Li Zhang, Ruihong Chen, Binyang Zheng, Xiangxi Wang, Sihong Xu, Ling Zhu, Kang Wang

**Affiliations:** a Key Laboratory of Infection and Immunity, Institute of Biophysics, Chinese Academy of Sciences, Beijing, China; b University of Chinese Academy of Sciences, Beijing, China; c Institute for In Vitro Diagnostics Control, National Institutes for Food and Drug Control, Beijing, China; d National Health Commission of the People’s Republic of China, Key Laboratory of Enteric Pathogenic Microbiology (Jiangsu Provincial Center for Disease Control and Prevention), Nanjing, China; Cornell University

**Keywords:** CVB5, viral evolution, cryo-EM structure, uncoating, antiviral strategy, viral survival

## Abstract

Coxsackie virus B5 (CVB5), a main serotype in human Enterovirus B (EVB), can cause severe viral encephalitis and aseptic meningitis among infants and children. Currently, there is no approved vaccine or antiviral therapy available against CVB5 infection. Here, we determined the atomic structures of CVB5 in three forms: mature full (F) particle (2.73 Å), intermediate altered (A) particle (2.81 Å), and procapsid empty (E) particle (2.95 Å). Structural analysis of F particle of CVB5 unveiled similar structures of “canyon,” “puff,” and “knob” as those other EV-Bs. We observed structural rearrangements that are alike during the transition from F to A particle, indicative of similar antigenicity, cell entry, and uncoating mechanisms shared by all EV-Bs. Further comparison of structures and sequences among all structure-known EV-Bs revealed that while the residues targeted by neutralizing MAbs are diversified and drive the evolution of EV-Bs, the relative conserved residues recognized by uncoating receptors could serve as the basis for the development of antiviral vaccines and therapeutics.

**IMPORTANCE** As one of the main serotypes in Enterovirus B, CVB5 has been commonly reported in recent years. The atomic structures of CVB5 shown here revealed classical features found in EV-Bs and the structural rearrangement occurring during particle expansion and uncoating. Also, structure- and sequence-based comparison between CVB5 and other structure-known EV-Bs screened out key domains important for viral evolution and survival. All these provide insights into the development of vaccine and therapeutics for EV-Bs.

## INTRODUCTION

Coxsackie virus B5 (CVB5) is a member of Enterovirus-B (EV-B) species, which can cause various human diseases, such as viral encephalitis; herpangina; hand, foot, and mouth disease (HFMD); aseptic meningitis; and acute flaccid paralysis (1–3). CVB5 infections have been common among infants and children in Asia, Europe, and south America in recent years (3–5), but there is no specific vaccine or antiviral therapy. Although studies on epidemics, phylogenesis, and pathogenesis of CVB5 have been extensively carried out, large gaps in the understanding of its structure and antigenicity still exist.

Similar to other EV-B species, CVB5 is a nonenveloped, positive-sense single-strand RNA (+ssRNA) virus (3, 6, 7). Its genome comprises 7,399 nucleotides that encode a 2,185-aa polyprotein, which is further proteolytically cleavaged into three segments: P1–3 (7, 8). P2, and P3 will break down into nonstructural proteins responsible for genome replication, whereas P1 is the precursor form of structural proteins VP1-VP4, which could be packaged into an icosahedral capsid (9). During the life cycle, mature full (F) viruses could attach to the cell surface when facing the specific receptors, which initiates a cascade of structural rearrangements and finally leads to the release of RNA genome into cytosol (10). After entry into the host cell, the viral RNA genome directs the gene replication and translation, producing proteins for virion assembly, based on which the protomers are packaged into procapsids. The procapsids are further matured by RNA packing followed by VP0 cleavage into VP2 and VP4, priming for the viral release through membrane fusion (11–17). During the life cycle, the viral capsid undergoes uncoating and maturation, serving as the direct targets for cell receptors and neutralizing MAbs (18, 19). Therefore, a detailed study of the capsid could aid in the understanding of viral cell entry, viral antigenicity, and pathogenesis, thus facilitating the development of antiviral vaccines and agents.

In this study, we solved the cryo-EM structures of CVB5 at atomic resolution. Structural analysis showed prominent features like the “canyon,” VP2 “puff,” and VP3 “knob” on the CVB5 F particle, as well as structural rearrangements on the terminal regions of VP1 and VP2 and the GH loop of VP3 responsible for the viral transition from F particle to A particle, reminiscence of classical features found in other EV-Bs, indicative of similar structural basis for antigenicity and cell entry. Also, important residues on EV-Bs having implications for viral infection and neutralization by MAbs were enumerated and fully analyzed. The results provide insights into the viral evolution and antiviral therapeutics development for EV-Bs.

## RESULTS

### Structure determination.

**FIG 1:**
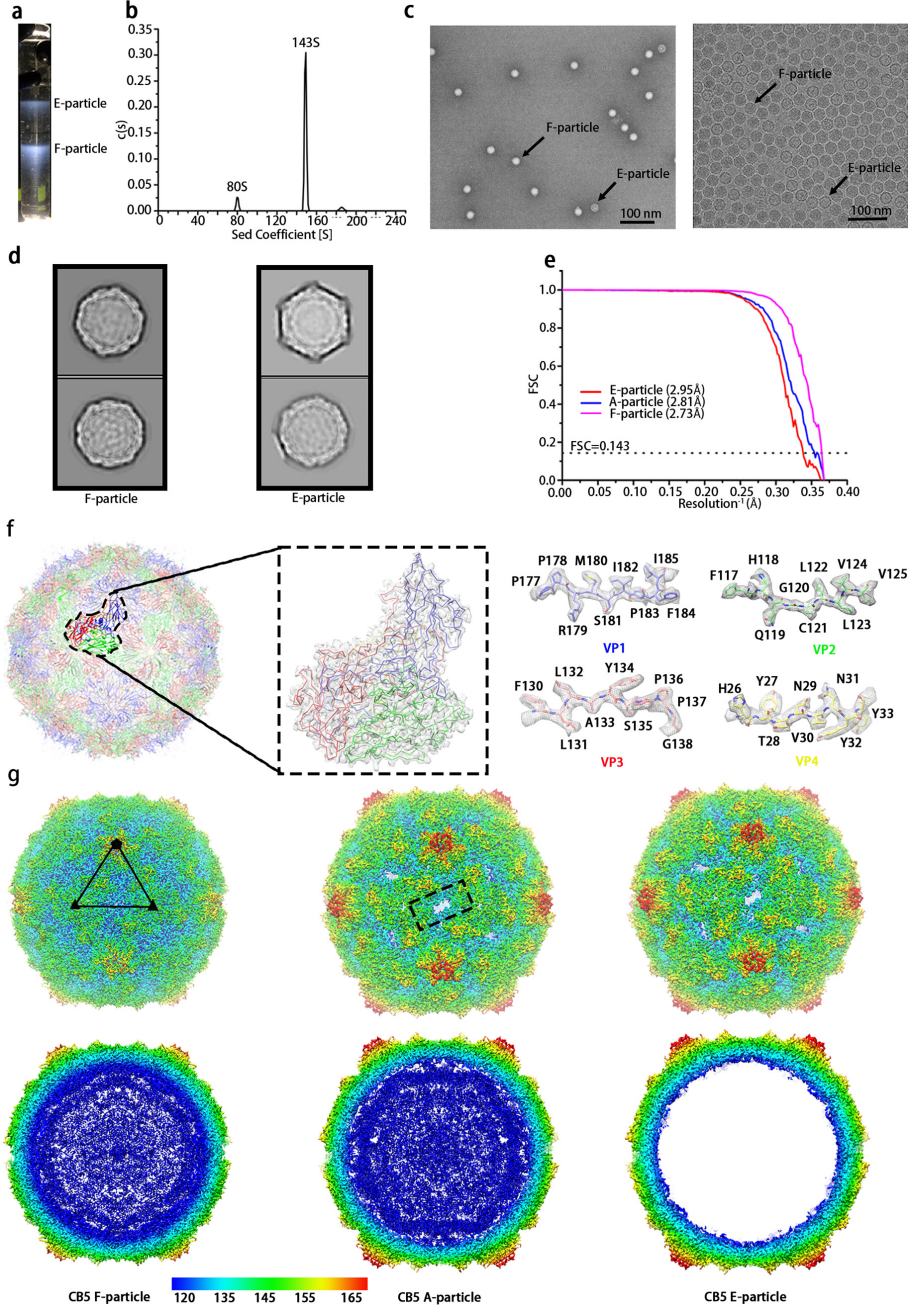
CVB5 purification and structure determination. (a) Sucrose density gradient concentration (15%-45%) for CVB5 purification, as described in Materials and Methods. Two obvious bands were observed, the upper band with an A260/A280 absorption ratio of 0.65 mainly containing E particle, and the lower band with an absorption ratio of 1.83 mainly containing F particle. (b) Analytical ultracentrifugation (AUC). The sample yielded two major peaks with sedimentation coefficients of 80 S and 143 S, respectively. (c) Negative staining image (left) and cryo-EM image (right) of F particle and E particle marked with arrows. (d) 2D classification of F (left) and E (right) particles in RELION 3.0. (e) Gold-standard Fourier Shell Correlation (FSC) curves of the final maps of CVB5 F (left), A (middle), and E (right) particles. (f) Cartoon representation of F particle from the view of 2-fold axis (left). One single zoomed-in protomer (middle) was shown in line and surface representations. The electron density maps from parts of VP1, VP2, VP3, and VP4 (right) were shown, respectively. VP1, VP2, VP3, and VP4 are colored in blue, green, red, and yellow, respectively. (g) Cryo-EM maps of F, A, and E particles were shown along the icosahedral 2-fold axis. The surface was colored by radius from blue to red corresponding to 120 nm to 165 nm.

CVB5 propagated in RD cells were first concentrated and purified via sucrose density gradient centrifugation, which indicated two bands in the tube (Fig. 1a). The samples corresponding to both the bands were pooled and mixed for analysis by analytical ultracentrifugation (AUC) (Fig. 1b), which suggested two types of particles–one containing RNA and the other lacking RNA with sedimentation coefficients of 143S and 80S, respectively. Consistently, the negative staining electron microscopy (EM) images also showed two types of particles (Fig. 1c). Based on what has been observed for Echovirus 30 (E30) and E3 (11, 18), the lower band (~143 S) might be the mixture of the F particles and A particles, which could not be distinctively separated out probably because of their similar molecular weights and mutual viscosity. The sample containing a mixture of viral particles was vitrified on a cryo-EM grid and loaded onto a 300-kV Titan Krios for data collection. In agreement with the results from AUC and EM, after data processing, three distinctive maps corresponding to F, A and E particles were reconstructed and refined to the resolutions of 2.73 Å, 2.81 Å, and 2.95 Å, respectively, according to the Fourier shell correlation at a threshold of 0.143 (Fig. 1d and e), which allowed an atomic model for the majority of the main chains and sides chains to be manually fitted or built in (Fig. 1f). All statistics on data collection and processing are summarized and given in Table 1. Compared with the map of the F particle, both maps of A and E particles had an expansion of ~4% with large openings on the 2-fold axes (Fig. 1g). However, no empty particle with a smaller radius was found after several rounds of 3D no-alignment classifications using different batches of particles, which is just like E18, CVB1 (13, 20). This is different from what has been found in the cases of E3, E30, E6, and E11, where an empty particle with a similar radius to the compacted F particle could be observed (11, 12, 15, 18, 21).

**TABLE 1:** Statistics for Cryo-EM imaging, data processing and refinement of models

Name	F-particle	E-particle	A-particle
Data collection			
Micrographs (total)	453	453	453
Micrographs (used)	452	452	452
Particles (used in final)	37080	14497	7675
Reconstructions			
Pixel size	1.35	1.35	1.35
Defocus range (μm)	1.2–2.5	1.2–2.5	1.2–2.5
Resolution (FSC = 0.143)	2.73	2.95	2.81
Model refinement			
Clashscore	8.49	8.93	6.69
Rotomer outliers (%)	0.14	0.29	0
C-beta deviations	0	0	0
Ramachandran statistics (%)			
Most favored	96.7	95.2	95.8
Allowed	3.21	4.49	3.99
Outliers	0.12	0.49	0.14
R.m.s. deviations			
Bond length (Å)	0.004	0.005	0.003
Bond angles (°)	0.548	0.613	0.522

### Structure characterization of the F particle.

**FIG 2:**
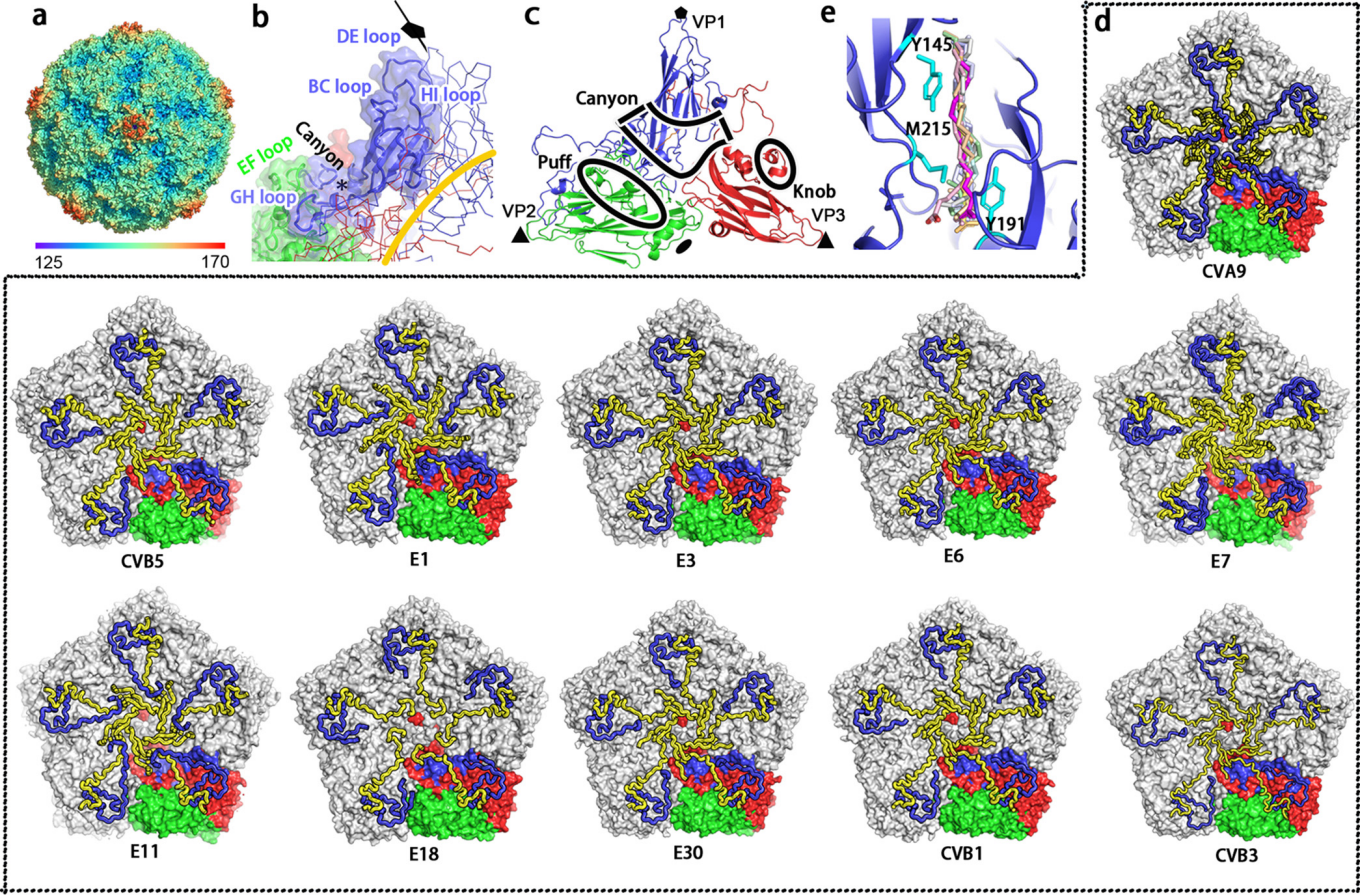
Structure features of CVB5 and other EV-Bs. (a) The surface representation of CVB5 F particle viewed down a 5-fold axis is colored by radius, as is depicted in the color bar below. Discontinuous shallower depressions known as canyons could be observed around the 5-fold axis. (b) Structure of the canyon region from a protomer. The canyon mainly formed by the VP1 BC loop, VP1 GH loop, and VP2 EF loop is shown in the cartoon (VP1, blue; VP2, green; VP3, red), and the hydrophobic pocket is marked with a black star. (c) Cartoon diagram of CVB5 F particle icosahedral asymmetric unit (VP1, blue; VP2, green; VP3, red), and the surface features of “canyon,” “puff,” and “knob” are indicated by the black line. (d) Inner surface representation of the penton from 11 structure-known EV-Bs like CVA9 (PDB code 1D4M), CVB5 (PDB code 7C9Y), CVB3 (PDB code 1COV), CVB1 (PDB code 7C9Z), E30 (PDB code 7C9S), E18 (PDB code 6HBG), E11 (PDB code 6LA3), E7 (PDB code 2X5I), E6 (PDB code 6ILP), E3 (PDB code 7C9X), and E1 (PDB code 1EV1). All VP4 and VP1 N termini are shown in loop and colored in blue and yellow, respectively. While four asymmetric units in a pentamer are colored in gray, one is colored in the signature colors (VP1, blue; VP2, green; VP3, red). (e) Close-up view of the CVB5 hydrophobic pocket. All pocket factors from the other 10 structure-known EV-Bs were aligned with CVB5 and are shown together with different colors, and residues interacting with pocket factors are shown in stick and colored in cyan.

As important features for EV-Bs, discontinuous shallower depressions, also known as the canyons, could be observed in CVB5 to circulate each 5-fold prominent cooling tower-like protrusion, which was constituted by the VP1 HI loops, DE loops, and BC loops of VP1 subunits (Fig. 2a). The canyon that has been demonstrated to serve as the structural basis for receptor binding site for enteroviruses was mainly formed by the VP1 BC loop, VP2 EF loop, and VP3 GH loop (11–13, 17, 22) (Fig. 2b). Another two more prominent loops exposed on the capsid surface of CVB5 were the so-called “puff” and “knob” structures, corresponding to part of the VP2 EF loop (residues 130–176 aa) and VP3 N-terminal helix (residues 54–70 aa) close to β-strand B, respectively (23) (Fig. 2c). These two structures have also been suggested to play an important role in the binding of both viral receptors and neutralizing antibodies for EV-Bs.

As expected, the icosahedral capsid of CVB5 F particle was made up of 60 copies of the four viral proteins VP1 to VP4. All these proteins were well ordered with the exception of some residues on the terminal regions of VP1 (1–9 aa at the N-terminus and 283 aa at the C-terminus) and VP2 (1–9 aa at the N-terminus and 260–261 aa at the C-terminus), as well as the residues from 14–24 aa on VP4. While the slender VP4 resided on the interior of the capsid shell, sneaking from the 5-fold axis to 3-fold axis, resembling all the other 10 structure-known EV-Bs including E1, E3, E6, E7, E11, E18, E30, CVB1, CVB3, and CVA9 (Fig. 2d) (11–13, 15, 18, 20, 21, 24–26), the other three viral proteins (VP1-3), which all contained the well-conserved eight-stranded anti-parallel β-barrel motifs, altogether constituted the outer surface of the capsid with their N termini located inside and C termini extended outward. Interestingly, most of the loops connecting the β sheets were exposed on the exterior of the surface and have been suggested to be involved in antibody and receptor binding for EV-Bs (Fig. 2c) (11, 18, 19, 23).

Besides, as important features for enteroviruses, a myristic acid moiety, was found to be covalently attached to the N-terminal glycine of the 69-amino-acid chain of VP4, a modification thought to play an essential role for viral assembly, maturation, and infectivity (11, 18). Also, a lipidic pocket factor that was modeled as PLM with an aliphatic chain of 16 carbon atoms based on its longer density was found to be lodged beneath the floor of the canyon in the center of VP1 (Fig. 2e), similar to other structure-known EV-Bs (Fig. 2e). The release of the pocket factor has been shown to be a necessary step to initiate uncoating and cellular infection for enteroviruses. Therefore, multiple anti-enterovirus compounds targeting the hydrophobic pocket to replace the pocket factor have been designed therefore to stabilize the capsids and prevent the virus from uncoating upon binding the cellular receptors (27–29).

### Structures of the A and E particles imply mechanism of particle expansion.

The procapsid E particle and uncoating intermediate A particle of CVB5 were structurally almost indistinguishable, each with an expansion of ~4% in capsid radius, compared with the compact mature F particle, which is different from E3 and E30, whose procapsid E particles were comparable to the F particle but smaller than the A particle (Fig. 1g) (11, 18).

**FIG 3:**
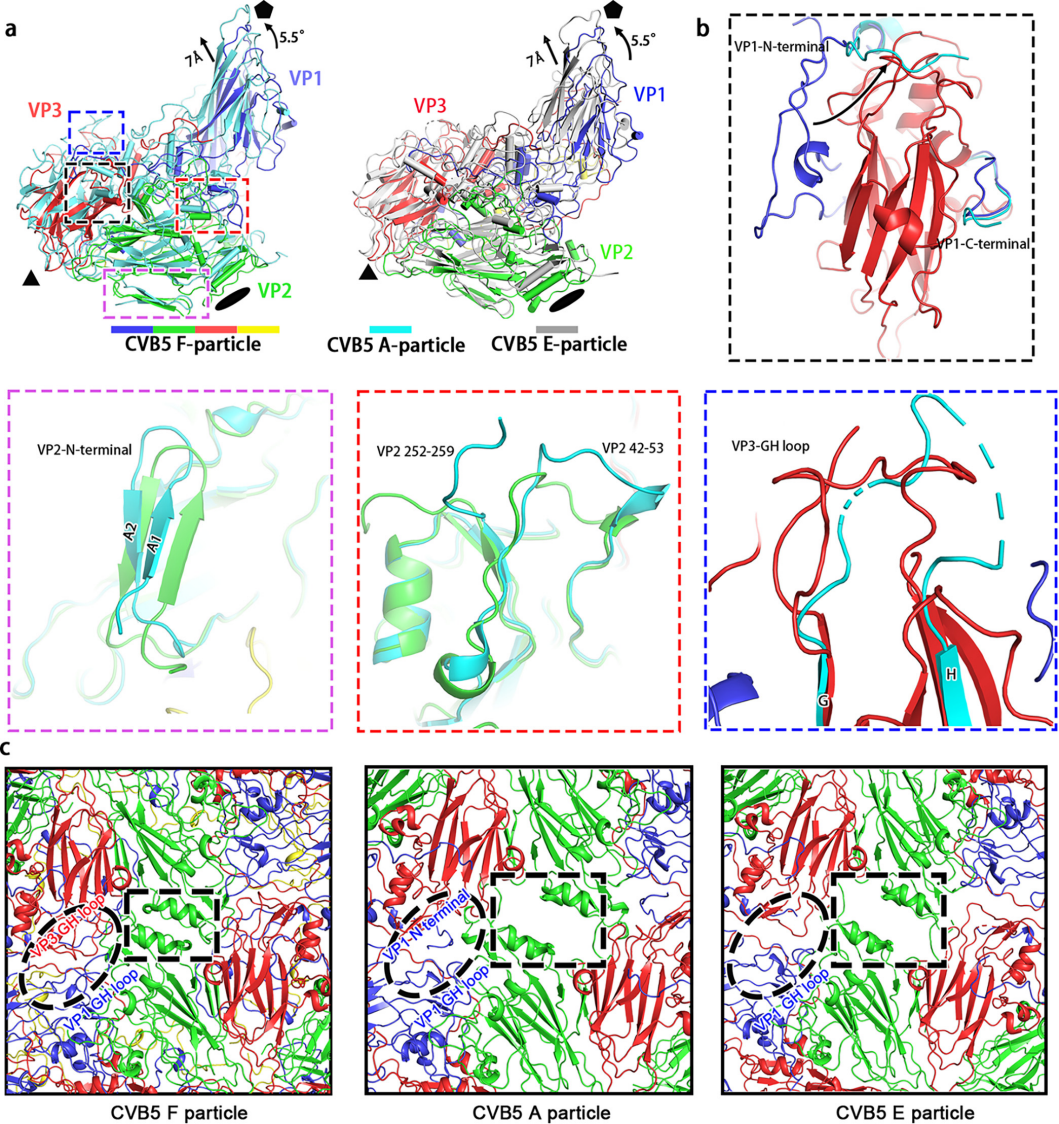
CVB5 particle expansion and uncoating. (a) Shown here are the superpositions of CVB5 F and A particles (left) as well as F and E particles (right) with respect to their icosahedral axes. Each viral protein of F particle is assigned the signature color as is shown in Fig. 1f; A particle and E particle are colored in cyan and gray, respectively. (b) Structural alterations during particle expansion. Structures of CVB5 F particle and A particle were superimposed and four domains exhibiting the most significant conformational changes were identified and shown in insets in different colors corresponding to four domains in (a) with boxes colored in the same color scheme. (c) Close-up views of F (left), A (middle), and E (right) particles on the 2-fold axis. The 2-fold axis channel highlighted in F-particle is closed, while the counterparts in A and E particles are open. The off-axis channels containing VP1 GH loop and VP3 GH loop are highlighted in ellipses.

Comparing the compact CVB5 F particle with the expanded A particle, we found that the expansion of CVB5 was largely attributable to the tectonic movements of viral proteins, accompanied by a ~5.5° counterclockwise rotation of the protomeric building unit pivoting about the 3-fold axis and a ~7 Å shift away of VP1 and VP3 from the particle center (Fig. 3a). This movement results in a more elongated structure and broadening of the surface mesa (Fig. 1e). Such a transition from F particle to A particle was also accompanied by the release of the pocket factor and internal festoon structure composed of VP4, disordering of the N-terminal portion (1–48 aa) of VP1, and a significant structural rearrangement of the terminal regions of VP1 (49–58 aa and 276–281 aa) and VP2 (12–31 aa, 42–53 aa, and 252–259 aa) (Fig. 3b). Furthermore, the whole GH loop of VP3 seems to shift from its position (Fig. 3b). We noted that the first seven residues of VP1 visualized on A particle would traverse the capsid and stop on the surface of the capsid, which was distinctive from the E particle where the corresponding residues were disordered, and all the other remaining residues were present on the interior of the capsid. Based on the previous studies on enteroviruses, it is reasonable to deduce that with the particle expansion, the GH loop of VP3 moved upward from the quasi-3-fold hole to form the classic β-hairpin structure to obstruct the off-axis channels and aid in the widening of the 2-fold helices (residues 90 to 98) connecting the C and D strands of VP2 (Fig. 3c). This rearrangement likely spared enough room allowing the extension of VP1 N-terminus to pass through the inner surface to reach the 2-fold gate and later slip into the quasi-3-fold hole (Fig. 3c), reminiscent of what has been proposed in other enteroviruses (i.e. EV71, E30, CVA16, and poliovirus) (11, 30–32). All these processes are suggested to be primed for the ensuing viral uncoating and genome release into the cytosol of infected cells.

### Structure and sequence analysis between EV-Bs.

**FIG 4:**
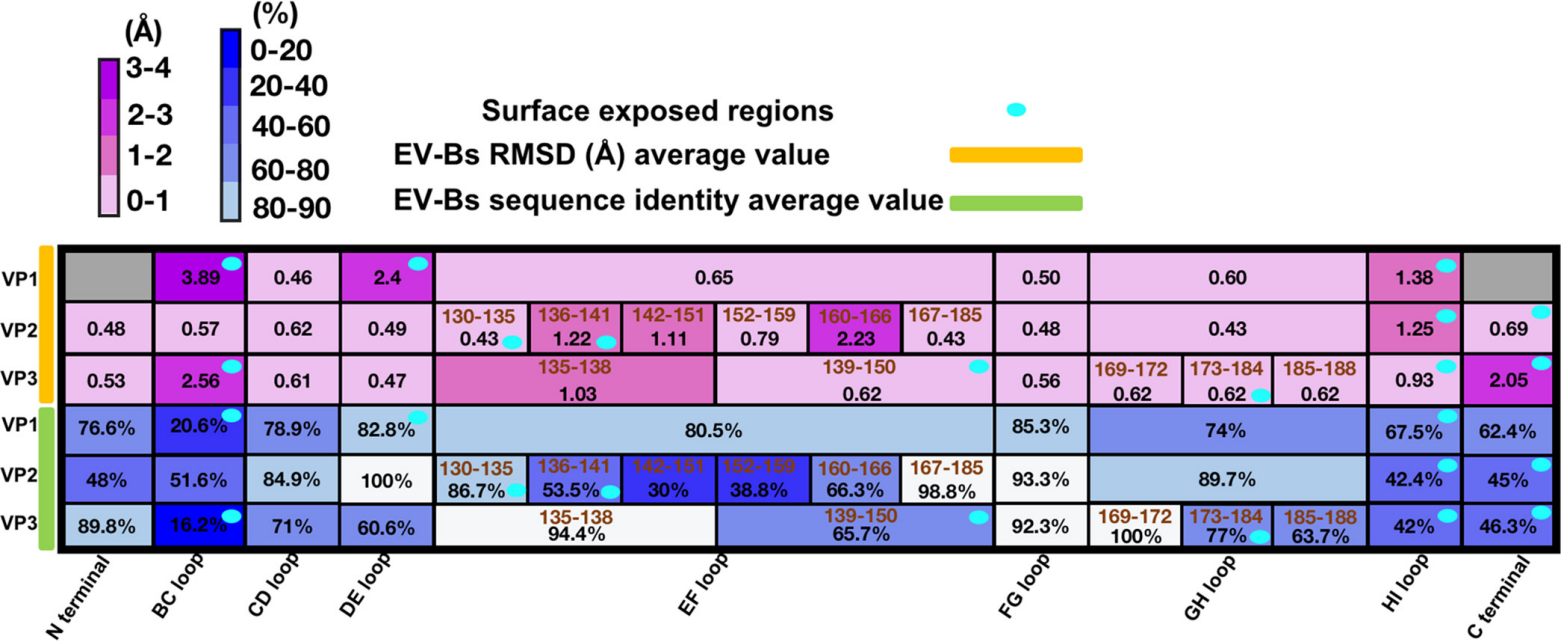
The comparison of 11 structure-known EV-Bs in sequence and structure. The RMSD value of each subdomain denotes the average of all RMSD values calculated by comparing CVB5 with each of the other 10 EV-Bs. Their corresponding sequence identities were the averages of all pairwise-based similarities of all these 11 EV-Bs in related subdomains. Heatmaps were generated based on the structure (top) and sequence (bottom) identities with calculated values (in black) present on the corresponding subdomains (in brown). The color bar of RMSD and sequence identity were displayed above; subdomains in gray represent data unobtainable because of the disordering of this region in some members. The surface-exposed regions are marked with ellipses colored in cyan.

Previous studies have suggested that the structural basis for receptor recognition by different EV-Bs is highly conserved. All the known EV-Bs to date use either FcRn or CAR as a receptor for uncoating and infection (11–13, 15, 17, 22, 24, 25). But the neutralization by different antibodies is commonly serotype-specific, though some epitopes of the neutralizing antibodies are structurally similar (18, 19). The difference in neutralization made us take a closer look at the structural and primary sequence features of different serotypes of EV-Bs. We compared the structures of CVB5 with the other 10 structure-known EV-Bs, all of which have been determined to an atomic level. While all the subunits except for VP4 follow the same β-barrel structure, they differ significantly in the N/C-terminuses and loops that connect the neighboring two β-strands, especially those exposed on the exterior of the capsid. The superimposition of each pair of the 11 structure-known EV-Bs showed that the average root-mean-square deviation (RMSD) in Cα positions of these EV-Bs on the surface-exposed regions of the VP1 BC loop (81–89 aa), VP1 DE loop (126–139 aa), VP1 HI loop (224–230 aa), VP2 EF loop (130–135 aa), VP2 EF loop (136–141 aa), VP2 HI loop (228–238 aa), VP2 C-terminus (254–261 aa), VP3 BC loop (74–81 aa), VP3 EF loop (138–150 aa), VP3 GH loop (173–185 aa), VP3 HI loop (200–206 aa), and VP3 C-terminus (231–238 aa) were 3.89, 2.41, 1.38, 0.43, 1.22, 1.25, 0.69, 2.56, 0.81, 0.62, 0.93, and 2.05 Å respectively. By contrast, the RMSD Cα positions of these EV-Bs on the VP1 EF loop (146–166 aa), VP1 FG loop (172–177 aa), VP1 GH loop (183–216 aa), VP1 CD loop (94–108 aa), VP2 BC loop (72–77 aa), VP2 CD loop (82–99 aa), VP2 DE loop (112–119 aa), VP2 EF loop (142–145 aa), VP2 EF loop (146–151 aa), VP2 EF loop (152–159 aa), VP2 EF loop (160–166 aa), VP2 EF loop (167–185 aa), VP2 FG loop (192–196 aa), VP2 GH loop (204–218 aa), VP3 CD loop (88–106 aa), VP3 DE loop (121–128 aa), VP3 EF loop (135–138 aa), VP3 FG loop (158–162 aa), and VP3 GH loop (169–172 aa), VP3 GH loop (185–188 aa), which reside on the interior of the capsid, were 0.65, 0.50, 0.60, 0.46, 0.57, 0.62, 0.49, 1.22, 1.11, 0.79, 2.23, 0.43, 0.48, 0.43, 0.61, 0.47, 0.81, 0.56, 0.62, and 0.62 Å respectively (Fig. 4). Collectively, the average RMSD on the exposed regions described above was 1.52 Å, which was relatively much larger than that of 0.71 Å, the average RMSD on the regions on the interior of the capsid. In agreement with this structural observation, the averaged sequence similarity among all the 11 EV-Bs on the interior regions was 78%, while it was only 53% for the domains that constitute the exterior regions.

## DISCUSSION

The infections caused by EV-Bs can result in aseptic meningitis and encephalitis, bringing about deep physical and mental distress and sorrow to the patients(1, 2). Although the symptoms are typically mild and highly self-limited for adults, they often give rise to severe disease and even death for fetus and infants. Plus, the ongoing evolution and potential recombination with other viruses would make possible emergence of highly pathogenic EV-Bs, posing a threat to public health. Here, we solved the structure of CVB5 and unveiled the key domains important for viral antigenicity and cellular entry for all EV-Bs.

Among the three forms of enterovirus particles, the compacted mature F particle is thought to be the only form that is capable of infecting host cells. Structures like the “canyon,” “puff,” and “knob,” which largely contribute to viral immunogenicity and cell entry, could be observed on CVB5, indicative of similar neutralizing and entry mechanism for CVB5 as those of other enteroviruses. By comparing the structure of CVB5 with all the other 10 structure-known EV-Bs, we characterized the domains that are important for viral entry, uncoating, and neutralization by antibodies. While the N-terminal residues of VP1 were disordered for most EV-Bs, they were visible in E1, E11, and CVA9 (15, 21, 24) and resided underneath VP4, albeit at slightly different directions, which is similar with enterovirus C and D (16, 33, 34). Importantly, similar to CVA16, an amphipathic helix, is present in the VP1 of all these 11 EV-Bs (30). As the amphipathic helix has been postulated to play an important role in particle expansion by associating with the cellular membrane (35, 36), all EV-Bs likely follow a similar structural rearrangement process during the early phase of viral uncoating, which is also supported by the structure of the 2-fold axis and off-axis channels of the CVB5 A particle, where the positions and orientations of the VP1/VP2 termini and VP3 GH loop are almost the same as observed for E30 and E3 (Fig. 3c). As another important factor for viral uncoating, the pocket factor could be found in all 11 EV-Bs, but it differs in the length of αC in different members. It has been modeled as DAO in E7 and E11 (15, 18), whereas it was modeled as the PLM in E1, E18, CVB1, and CVB5 owing to the longer density of αC (13, 20, 24). As the case for E3, E30, and E6, where the density seems longer than PLM, it was annotated as the molecule of SPH (11, 12, 18). However, it is noteworthy that the residues that directly interact with the pocket factors are highly conserved across all the 11 EV-Bs (Fig. 2e). These residues might serve as precise targets for the design of universal antiviral agents since the release of a pocket factor always leads to the collapse of pocket structure, which is an essential step for viral uncoating and infection. Besides, all structure-known EV-Bs except E18 have a myristoylation on the VP4 N-terminus (20). In many structures it is not clear whether the myristoylation is present or not since the VP4 N terminus is disordered (16, 37). But by analogy with other enteroviruses (11, 12, 15), the presence of pocket factor and myristoylistic acid in the mature F particle but not immature E particle and intermediate A particle, suggests that both the two molecules play important roles in the stabilization and integrity of mature virions for most of the EV-Bs.

**FIG 5:**
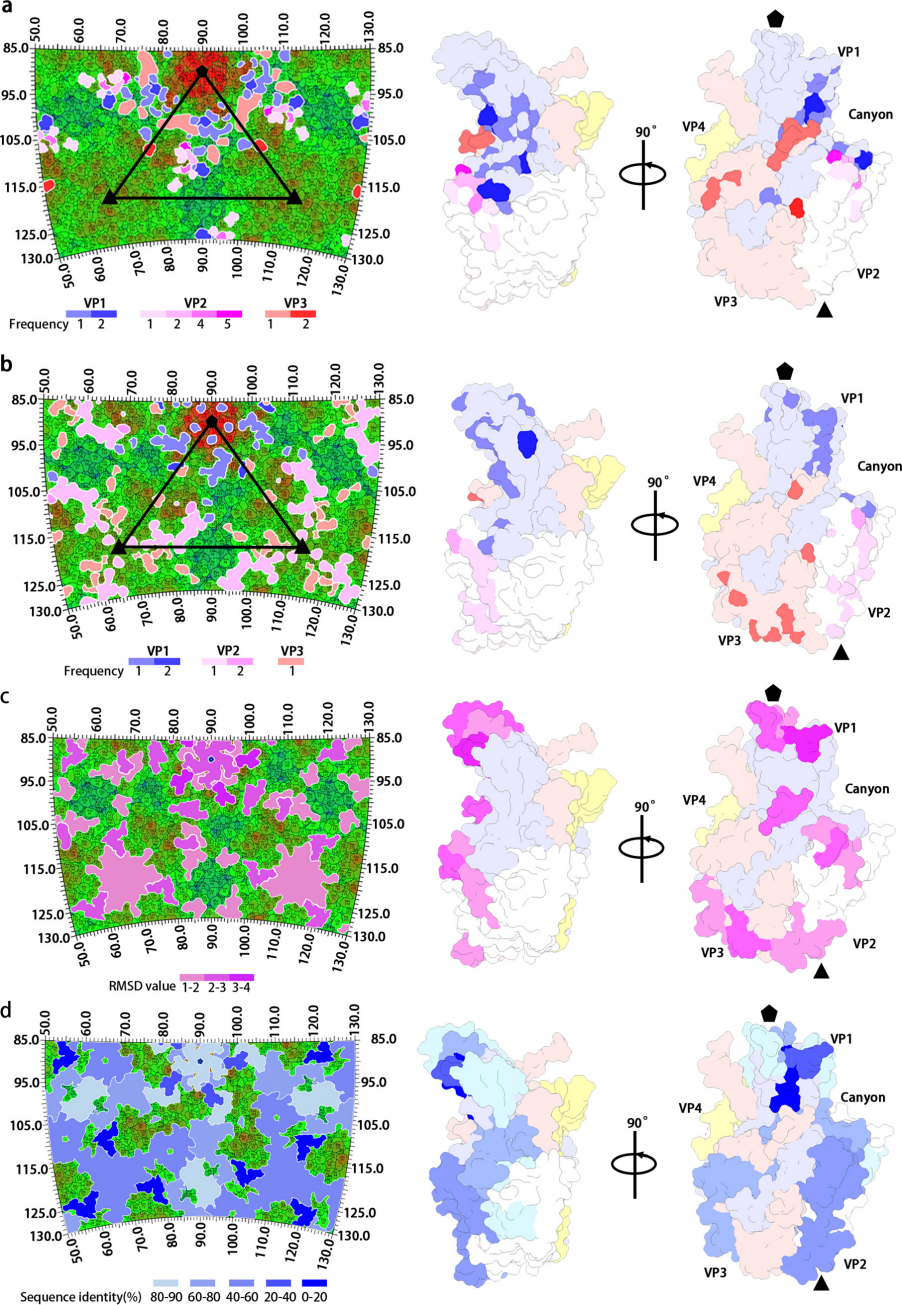
Features of antibody and receptor binding sites on EV-Bs. Footprints of (a) EV-B functional receptors (FcRn and CAR) and (b) all published EV-B-specific neutralizing MAbs are colored in frequency of interactions, as is shown in color bars below and are exhibited on the CVB5 roadmap (left) and asymmetric unit (right). Diversity profiles of subdomains on EV-Bs with RMSD (c) and sequence identity (d) gradients shown below corresponding to Fig. 4 are displayed on the CVB5 roadmap (left) and asymmetric unit (right). All CVB5 stereographic projection roadmaps here are colored in radius from blue (120 nm) to red (165 nm). All CVB5 asymmetric units are shown in two different orientations with VP1, VP2, VP3, and VP4 colored in light blue, white, light pink, and light yellow, respectively.

Previous studies have suggested that the engagement between enterovirus and its corresponding uncoating receptor is largely determined by the viral structure (11–13, 15). Different enteroviruses could recognize the same receptor molecule if they share the similar canyon structure, which is largely constituted by VP1 BC loop, VP2 EF loop, and VP1 GH loop. The structure of these canyon-related loops could also be used for the prediction of uncoating receptor of a given virus (11). We summarized the viral residues of EV-Bs that are involved in the binding with uncoating receptors, including 2 on VP1 BC loop, 2 on VP1 βC, 2 on VP1 CD loop, 5 on VP1 EF loop (148–150, 154–155), 7 on VP1 GH loop, 2 on VP1 βH, 2 on VP1 C terminus (260 and 262), 12 on VP2 EF loop, 1 on VP2 C terminus, 3 on VP3 GH loop (180, 182–183), and the last residue of VP3 (Fig. 5a). As expected, 92% of residues are located inside or in the vicinity of the canyon structure, and they are relatively conserved in structure across all the 11 structure-known EV-Bs, as is shown in Fig. 4 and 5a. On the other hand, we also listed the viral residues targeted by neutralizing MAbs specific to EV-Bs. Of these immunogenic dominant residues, 1 is on VP1 βB; 6 are on VP1 BC loop, 2 are on βC, 1 is on VP1 DE loop, 2 are on VP1 EF loop, 1 is on VP1 βH, 1 is on VP1 HI loop, 6 are on VP1 C-terminus, 1 is on VP2 βB, 2 are on VP2 BC loop, 12 are on VP2 EF loop, 4 are on VP2 HI loop, 4 are on VP3 BC loop, 2 are on VP3 EF loop, 1 is on VP3 βI, and 1 is on VP3 C-terminus. Seventy-nine percent of the residues are located outside of the canyon and are less conserved in sequence, with some being highly varied in structure (Fig. 4, 5b, c and d). These observations suggested that most of the neutralizing MAbs against EV-Bs might be serotype-specific, similar to what has been observed in E30 and E3, because the variations in epitope usually lead to the abolishment of neutralizing antibodies against viruses. While most of the residues that interact with the receptors are themselves part of the canyon, the epitope residues targeted by MAbs are distributed in the regions around the rims of canyon. Therefore, the evolution and emergence of new serotypes of enteroviruses are probably driven by the immunity imposed on them. To evade the immune pressure of highly potent neutralizing antibodies, a virus needed to keep mutating to make these antibodies ineffective. The mutations on these sites outside of the canyon for EV-Bs seemed to take the priority for viral survival. When beneficial mutations are accumulated enough to abolish almost all the binding activity of neutralizing antibodies, the virus evolves into a new serotype. But for some viruses, mutations in canyon might result in the abolishment of binding with its receptor. Thus, a lower frequency of mutations, especially those that would have impact on the canyon structure, could be observed. Moreover, mutations in the vicinity of canyon are rare, which in some limited circumstances might result in the change of uncoating receptor and evolve to a new species of Enterovirus. Therefore, the development of a universal antiviral agent or vaccine against EV-Bs should be focused more on these residues associating with receptors rather than those interacting with antibodies.

In summary, we described here the high-resolution structure of CVB5 and gave evidence for its antigenicity and uncoating mechanism. Together with the information from structural comparison, we tried to pinpoint amino acids inside or in the vicinity of the canyon of EV-Bs that might drive the evolution of EV-Bs and also amino acids outside of the canyon, which could be taken into consideration while designing universal vaccines and therapeutics effects against EV-Bs.

## MATERIALS AND METHODS

### CVB5 production and purification.

CVB5 strain isolated from Jiangsu Provincial Center for Disease Control and Prevention (CDC) was inoculated onto human rhabdomyosarcoma (RD) cell monolayers at a multiplicity of infection (MOI) of 0.01, which were cultured in Dulbecco’s modified Eagle’s medium (DMEM; Sigma) with 2% fetal bovine serum (FBS; Gibco). After incubated at 37°C for 48h, the cultures were harvested and then treated with freeze–thaw cycles three times for cells lysis. In order to facilitate virus release from the cells, 1% NP-40 was added into PBS (pH 7.4). Then the solution was centrifuged at 1,500 × *g* at 4°C for 30 min to remove cell debris, and the supernatant was ultra-centrifuged at 12,000 × *g* at 4°C for 2h. The harvested pellets were resuspended in PBS overnight and loaded onto continuous 15–45% (wt/vol) sucrose density gradient before centrifuging at 104,100 × *g* at 4°C for 5h. Two sets of fractions were collected and dialyzed against PBS buffer.

### Negative staining electron microscope.

The purified CVB5 samples were diluted into a concentration about 0.5 mg/mL with PBS (pH 7.4) buffer. Five μL sample was loaded onto a glow discharged carbon support film and stained with 1% phosphotungstic acid (pH 7.0) for 1 min and then loaded onto the FEI Tecnai Spirit 120-kV TEM for imaging.

### Cryo-EM and data collection.

Aliquots (3 μL) of purified samples of E-particle or F-particle were deposited onto glow discharged holey carbon Quantifoil Cu grid (R 1.2/1.3, 400 mesh, Quantifoil Micro Tools) into FEI Vitrobot at 25°C with a humidity level of 100%. The grid was blotted for 3 s before plunge-frozen into liquid ethane cooled by liquid nitrogen and then examined under low dose conditions with a Titan Krios electron microscope 300 kV (FEI). All movies (25 frames, each 0.2 s, total dose of 30 e-/Å^2^) were collected on a Gatan K2 Summit detector with defocus ranging between 1.2 and 2.5 μm and at a nominal magnification of 59,000 corresponding to a pixel size of 1.35.

### Cryo-EM single particle 3D reconstruction.

The 3D reconstruction process was performed in RELION 3.0 (38). A total of 453 micrographs were selected for beam induced motion correction with Motion Cor2 procedure. The contrast transfer function (CTF) parameters of each micrograph were estimated by the Gctf program (39). Particles were picked automatically by Laplacian-of-Gaussian method, and the false positives were removed manually. Finally, a total of 84,730 particles were extracted for 2D classification. We can detect full-particle (F-particle) and empty-particle (E-particle) according to the existence of nucleic acid signals from 2D averaged particles. Then particles were extracted for *ab initio* 3D model generation, three-dimensional auto-refinement, and three-dimensional no alignment classification, generating the final models of full particle, empty particle, and A-particle by using 37,080, 14,497, and 7,675 particles, respectively. After the high-resolution reconstruction, the final resolution was evaluated on the basis of the gold-standard Fourier shell correlation (FSC threshold = 0.143).

### Model building and refinement.

The structure of CVB3 (Protein Data Bank [PDB] ID: 1COV) was manually fitted into the map of CVB5 full particle using Chimera and mutated to the amino acid sequence of CVB5 in COOT (40). The atomic model was refined by real-space refinement procedure in Phenix (41) and readjusted in COOT, and then the process above was repeated several times afterwards. Structures of CVB5 empty particle and A-particle were solved using the same strategy.

### Analytical ultracentrifugation (AUC).

Sedimentation velocity experiments were performed on a Beckman XL-I analytical ultracentrifuge at 20°C. Samples prepared at high concentration were diluted with PBS buffer (pH 7.4) to 400 μL with A280 nm absorption of about 0.7 and further loaded into a conventional double-sector quartz cell and then mounted in a Beckman four-holeAn-60Ti rotor. Data were collected at 8064 × *g* at a wavelength of 280 nm. Finally, the interference sedimentation coefficients were calculated using the SEDFIT software program (sedfitsedphat.nibib.nih.gov/software).

### Data availability.

The atomic structures of F-, E-, A-particles have been submitted to the Protein Data Bank with accession codes 7C9Y, 7WL3, and 7XB2, respectively.
